# Expression of MAGE-C1/CT7 and MAGE-C2/CT10 Predicts Lymph Node Metastasis in Melanoma Patients

**DOI:** 10.1371/journal.pone.0021418

**Published:** 2011-06-27

**Authors:** Alessandra Curioni-Fontecedro, Natko Nuber, Daniela Mihic-Probst, Burkhardt Seifert, Davide Soldini, Reinhard Dummer, Alexander Knuth, Maries van den Broek, Holger Moch

**Affiliations:** 1 Division of Internal Medicine and Clinic of Oncology, University Hospital, Zurich, Switzerland; 2 Institute for Surgical Pathology, University Hospital, Zurich, Switzerland; 3 Biostatistics Unit, Institute of Social and Preventive Medicine, University of Zurich, Zurich, Switzerland; 4 Clinic of Dermatology, University Hospital, Zurich, Switzerland; University of Texas Southwestern Medical Center at Dallas, United States of America

## Abstract

MAGE-C1/CT7 and MAGE-C2/CT10 are members of the large MAGE family of cancer-testis (CT) antigens. CT antigens are promising targets for immunotherapy in cancer because their expression is restricted to cancer and germ line cells and a proportion of cancer patients presents with immune responses against CT antigens, which clearly demonstrates their immunogenicity. This study investigates the expression of MAGE-C1/CT7 and MAGE-C2/CT10 in primary and metastatic melanoma. Immunohistochemical staining of tissue microarrays that consisted of 59 primary malignant melanomas of the skin, 163 lymph node and distant melanoma metastases and 68 melanoma cell lines was performed. We found MAGE-C1/CT7 expression in 15 out of 50 (24%) primary melanomas and 15 out of 50 (24%) cell lines, whereas MAGE-C2/CT10 was detected in 17 out of 51 (33%) primary melanomas and 14 out of 68 (17%) cell lines. MAGE-C1/CT7 and MAGE-C2/CT10 were both detected in 40% of melanoma metastases. Patients with MAGE-C1/CT7 or MAGE-C2/CT10 positive primary melanoma had significantly more lymph node metastases (p = 0.005 and p<0.001, resp.). Prediction of lymph node metastasis by MAGE-C1/CT7 and MAGE-C2/CT10 was independent of tumor cell proliferation rate (Ki67 labeling index) in a multivariate analysis (p = 0.01). Our results suggest that the expression of MAGE-C1/CT7 and MAGE-C2/CT10 in primary melanoma is a potent predictor of sentinel lymph node metastasis.

## Introduction

Melanoma is an aggressive malignant disease with poor prognosis and its incidence is increasing faster than any other cancer. Patients with metastatic disease have a median survival of nine months and a five-year survival below 15% [1]. Melanoma treatment is an ongoing issue in clinical oncology: For early stage of disease, surgical excision remains the best treatment option whereas adjuvant therapy is not broadly indicated due to unfavorable risk–benefit ratios [2]. In advanced stage of disease, resistance to standard chemotherapy frequently occurs [3], [4] and standard immunotherapy shows only moderate success [5] but recently promising data have been shown by blockade of T cell regulatory molecules [6]. However, spontaneous, complete regression of melanoma sporadically occurs [7], which presumably is mediated by cancer-specific immunity [8], [9], [10] and thus suggests that improvement of immunotherapeutical approaches is worth pursuing. In any case, identification of patients having a high risk of melanoma metastatic spread at the time of diagnosis is crucial in order to detect the subset of patients most likely to benefit from strict follow-up.

Cancer-testis (CT) antigens represent a family of proteins widely studied in the field of cancer immunotherapy because of their restrictive expression pattern and immunogenicity in cancer patients [11]. In normal tissues, the expression of CT antigens is restricted to germ line tissues (namely placenta, ovaries and testis), which express small amounts of HLA molecules. In malignant tissues, the expression of CT antigens is highly erratic with frequent co-expression of several CT antigens. Few studies demonstrated that CT antigen expression correlates with tumor growth, survival and relapse of disease [12]. Among CT antigens, the MAGE family is one of the most extensively investigated so far, with documented expression in several cancers. MAGE-C1/CT7 has been simultaneously identified by representational difference analysis in a melanoma cell line [13] and its immunogenicity assessed by serological analysis of recombinant cDNA expression libraries (SEREX) [14] from a melanoma patient. Subsequently, several studies showed MAGE-C1/CT7 to be highly expressed in a variety of other human cancers [13], [15]. Recently, we demonstrated that MAGE-C1/CT7 spontaneously induces a specific cellular immune response in melanoma patients [16], suggesting MAGE-C1/CT7 as a potential target for melanoma immunotherapy. However, data concerning MAGE-C1/CT7 protein expression in melanoma are limited [15], [17]. MAGE-C2/CT10 is another MAGE antigen with high similarity to MAGE-C1/CT7 discovered by representational difference analysis from a melanoma cell line [18]. So far, MAGE-C2/CT10 expression in melanoma lesions has only been analyzed by RT- PCR [18] and not on the protein level. Its potential role to evoke broad humoral and cellular immune responses has been shown *in vivo* in melanoma patients [18], [19], [20]. In various tumor types, the expression of MAGE-C2/CT10 and MAGE-C1/CT7 varied considerably [15], [21]. Consequently, we investigated the expression of MAGE-C1/CT7 and MAGE-C2/CT10 protein on tissue microarrays comprising of 222 primary and metastatic melanoma lesions as well as 68 melanoma cell lines by immunohistochemistry.

## Materials and Methods

### Western blot and recombinant proteins

Recombinant MAGE-C1/CT7 and MAGE-C2/CT10 were produced and used in western blot analysis as previously described [22], [23].

### Patient's samples and cell lines

222 melanoma lesions, including 68 primary malignant melanoma and 163 lymph node and distant metastases of different anatomic sites (114 brain, 24 lymph node, 7 soft tissue, 5 gastrointestinal, 4 skin, 4 lung, 2 liver, 1 parotid, 1 bone and 1 adrenal metastases) were analyzed on tissue microarrays (TMA). Primary malignant melanomas were derived from a clinical sentinel lymph node (SLN) trial in melanoma patients [24]. Clinical data and SLN biopsies were available for 51 of 59 primary melanomas. Tissue of melanoma metastases was identified in the archives of the Institute for Surgical Pathology Zurich. In addition, cell lines derived from 68 melanoma metastases obtained from surgical specimen of melanoma patients were analyzed. This cohort of melanoma cell lines and melanoma metastases was previously reported [25]. All patients were admitted at the University Hospital of Zurich and provided written informed consent in accordance with the Declaration of Helsinki. The Ethical Commission Zurich, Switzerland, approved the here reported lymph node study (approval number Stv. 16-2007).

### Tissue microarray (TMA)

A morphologically representative region from paraffin-embedded tissue of 68 primary melanomas and 163 melanoma metastases was used for the construction of 4 TMAs. A core tissue biopsy (diameter 0.6 mm) from the donor paraffin block was used and precisely arrayed into a new recipient paraffin block using a costumer built instrument [26]. A cell line TMA was constructed as recently described [25].

### Immunohistochemistry and immunofluorescence

4.0 µm sections were cut, mounted on glass slides, deparaffinised, rehydrated and stained with hematoxylin-eosin using standard histological techniques. For immunohistochemical staining, the Ventana Benchmark automated staining system and Ventana reagents were used (Ventana Medical Systems, Tucson, AZ). Immunohistochemistry and immunofluorescence were performed as recently described [21], [27]. Primary antibodies against MAGE-C1/CT7 (clone CT7-33, Dako Cytomation, Dilution 1∶80, Glostrup, Denmark), against MAGE-C2/CT10 (clone LX-CT10.5, Dilution 1∶100 [28] and against Ki67 (Ki67, clone MIB-1, Dilution 1∶20, Dako Cytomation, Glostrup, Denmark) were used. Immunohistochemical labeling for MAGE-C1/CT7 and MAGE-C2/CT10 in equal or more than 5% of melanoma cells was recorded as positive; this cut-off has been previously reported [15], [17]. The Ki-67 Labeling Index (LI) is calculated as the number of positive nuclei per 100 melanoma cells was recorded.

### MAGE-C1/CT7-specific RT-PCR

In order to confirm the positivity of MAGE-C1/CT7 detected by IHC and IFC RT-PCR was performed using RNA samples extracted from melanoma cell lines which were included in the above mentioned TMA. Melanoma cell lines were either expressing MAGE-C1/CT7 in the cytoplasm (M01.0119), in both nucleus and cytoplasm (M990514, M980409, M01326) or were negative for MAGE-C1/CT7 (M950504, M010817). Cell lines were derived from metastatic melanoma lesions and established in the Department of Dermatology, University Hospital Zurich, Switzerland. Total RNA was extracted by the Qiagen RNA Extraction Kit (Qiagen, Hombrechtikon, Switzerland) and RNA was reverse-transcribed into cDNA using the TaqMan EZ RT-PCR kit (Applied Biosystems, Rotkreuz, Switzerland) according to the manufacturer's instructions. For PCR, cDNA was diluted in TaqMan universal PCR master mix supplemented with a MAGE-C1/CT7-specific TaqMan probe and MAGE-C1/CT7-specific forward (5′-GACGAGGATCGTCTCAGGTCAGC-3′) and reverse (5′-ACATCCTCACCCTCAGGAGGG-3′) primers (all from Applied Biosystems, Rotkreuz, Switzerland).

### Statistical analysis

Categorical data were compared between groups using chi-square test or Fisher's exact test where appropriate. Continuous data were compared using the Mann-Whitney test. A logistic regression was performed to evaluate the predictive power of Ki-67 LI and MAGE-C1/CT7 expression in primary malignant melanoma for lymph node metastasis. Statistical analyses were performed using SPSS 13.0 (SPSS Inc, Chicago IL). Multivariable exact logistic regression was performed using Stata 10.0 (StataCorp, Lakeway Drive, TX) to analyze predictive power of positive MAGE-C1/CT7 or MAGE-C2/CT10 adjusted for Ki-67 proliferation rate (Ki-67 LI). p-values <0.05 were considered significant.

## Results

### MAGE-C1/CT7 and MAGE-C2/CT10 expression

**Figure 1:**
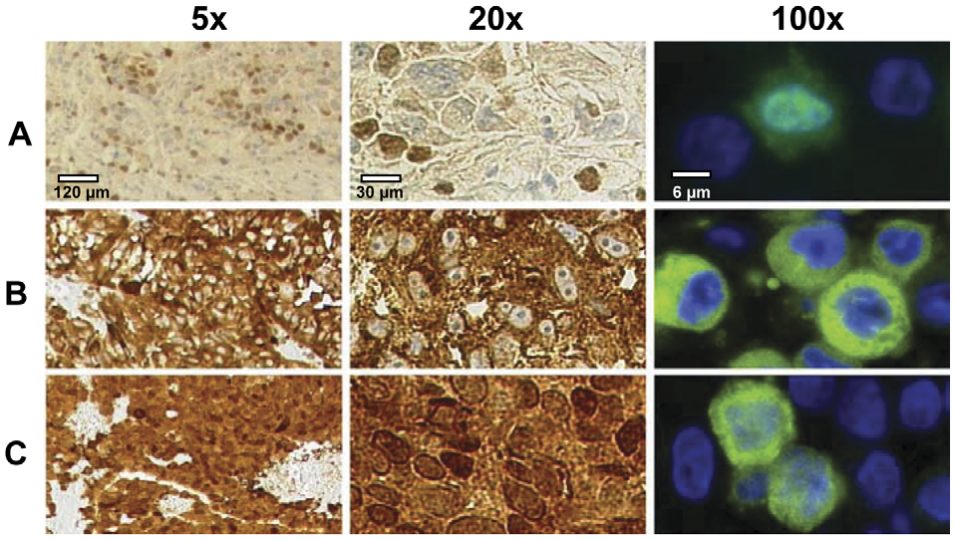
MAGE-C1/CT7 protein expression pattern in melanoma. MAGE-C1/CT7 expression was analyzed by immunohistochemistry on melanoma lesions at two different magnifications (5x, 20x) (left and middle panels) and by immunofluorescence on cell lines (100x) (right panels). Expression of MAGE-C1/CT7 is shown only in the nucleus (A), only in the cytoplasm (B) and both in nucleus and cytoplasm (C).

**Figure 2:**
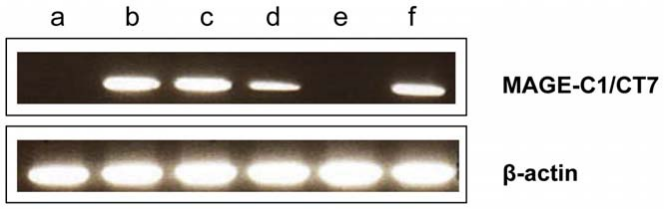
MAGE-C1/CT7 RNA expression in cell lines. MAGE-C1/CT7 RNA expression was analysed on the following cell lines: (a) M950504, (b) M980409, (c) M010119, (d) M990514, (e) M01817 and (f) M010326 melanoma cell lines; (a) and (e) did not express MAGE-C1/CT7; (b), (c), (d) and (f) were positive. Beta actin control is shown.

Antibodies specific for MAGE-C1/CT7 and MAGE-C2/CT10 were confirmed on WB with recombinant proteins (Fig. S1). MAGE-C1/CT7 was found in the nucleus or cytoplasm of melanoma cells. Most cases showed a combined nuclear and cytoplasmatic (n = 10) or only a cytoplasmatic (n = 4) MAGE-C1/CT7 expression (Fig. 1A, B or C). The MAGE-C1/CT7 expression pattern was further analyzed by immunofluorescence staining for MAGE-C1/CT7 and for a nuclear marker (DAPI). Only one primary melanoma and one melanoma cell line were characterized by an exclusive nuclear MAGE-C1/CT7 expression. RT-PCR confirmed MAGE-C1/CT7 expression in all cell lines, independently of the sub-cellular expression pattern and we therefore scored both expression patterns as positive (Fig. 2). We observed MAGE-C1/CT7 expression in 15 of 50 cell lines (24.2%), 12 of 59 primary melanomas (20.3%), and 66 of 163 metastases (40.5%) in the TMAs (Table 1).

**Table 1:** MAGE-C1/CT7 protein expression in melanoma biopsies from primary, metastatic lesions and melanoma cell lines.

		CT7 Positive
	N	N (%)
**Cell lines**	50	15 (24)
**Primary melanoma**	59	12 (20)
**Metastases**	163	66 (40)

MAGE-C1/CT7 expression was evaluated using tissue microarrays (TMAs) consisting of 50 melanoma cell lines, 59 primary melanoma lesions and 163 metastases. Expression was considered positive if at least 5% of the tumor cells expressed MAGE-C1/CT7.

**Figure 3:**
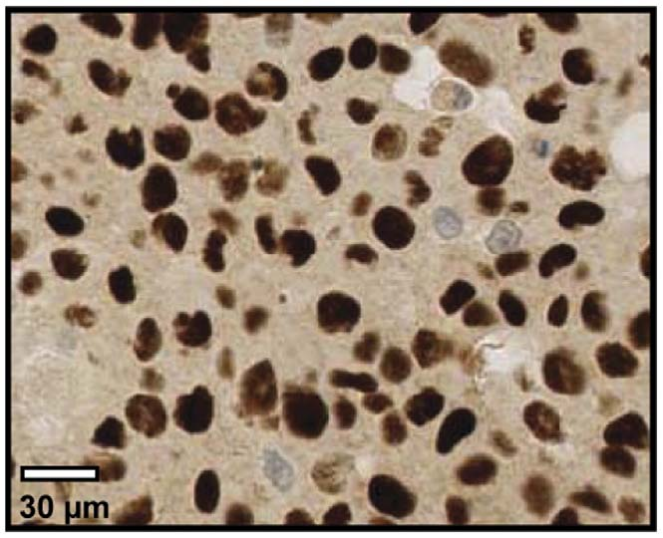
MAGE-C2/CT10 protein expression in melanoma. MAGE-C2/CT10 nuclear expression analyzed by immunohistochemistry from a metastatic melanoma lesion (20X).

In contrast to MAGE-C1, MAGE-C2/CT10 expression was only found in the nucleus (Fig. 3). MAGE-C2/CT10 expression was identified in 14 of 68 cell lines (20%), in 17 of 51 primary melanomas (33%), and in 62 of 155 metastases (40%) (Table 2). Due to technical reasons, biopsies from 8 primary lesions and 8 metastases from the original TMA could not be analyzed for MAGE-C2/CT10. The expression of these two antigens significantly correlated with each other (p<0.001). Co-expression was detected in 9 out of the total 51 (18%) primary lesions and 36 out of total 137 (26%) metastases.

**Table 2:** MAGE-C2/CT10 protein expression in melanoma biopsies from primary, metastatic lesions and melanoma cell lines.

		CT10 Positive
	N	N (%)
**Cell lines**	68	14 (20)
**Primary melanoma**	51	17 (33)
**Metastases**	155	62 (40)

MAGE-C2/CT10 expression was evaluated using tissue microarrays (TMAs) consisting of 68 melanoma cell lines, 51 primary melanoma lesions and 155 metastases. Expression was considered positive if at least 5% of the tumor cells expressed MAGE-C2/CT10.

### MAGE-C1/CT7 and MAGE-C2/CT10 expression and correlation with sentinel lymph node metastasis

The availability of primary melanoma from a SLN study [24] allowed us to directly analyze the relevance of MAGE-C1/CT7 and MAGE-C2/CT10 expression for lymph node metastasis. MAGE-C1/CT7 positive primary melanomas had significantly more frequent SLN lymph node involvement (pN1) than negative ones (44% vs. 3%; p = 0.001; Table 3A) with a positive predictive value of 87%. Similarly, MAGE-C2/CT10 positive primary melanomas showed significantly more lymph node involvement (pN1) than negative melanomas (58% vs. 8%; p = 0.001; Table 3B) with a positive predictive value of 80%.

**Table 3:** Correlation between MAGE-C1/CT7 (A) and MAGE-C2/CT10 (B) expression in primary melanoma and lymph node metastases.

A			
pN Stage	N	CT7 Positive N(%)	p value
**pN1**	16	7 (44)	0.001
**pN0**	35	1 (3)	

B			
pN Stage	N	CT7 Positive N(%)	p value
**pN1**	17	10 (58)	<0.001
**pN0**	34	3 (8)	

Primary melanoma with sentinel lymph node (SLN) involvement (pN1) and primary melanoma without SLN involvement (pN0). The correlation was considered significant by a p-value ≤0.05.

We described in a previous study that the tumor cell proliferation rate as measured by the Ki-67 Labeling Index significantly correlates with the presence of lymph node metastases (p = 0.03) in univariate analysis [24] (Table 4A). A multivariable exact logistic regression analysis revealed that MAGE-C1/CT7 as well as MAGE-C2/CT10 expression in the primary lesions are independent predictors of metastases when adjusted for Ki-67 (p = 0.005 and p<0.01 resp.), whereas Ki-67 is not an independent predictor (p = 0.29; Tab. 4A and p = 0.3 Tab. 4B resp.). Both MAGE-C1/CT7 and MAGE-C2/CT10 remain independent predictors when analyzed together by a multivariate analysis even if adjusted for each other (p = 0.03 und p = 0.01 resp.) (Table 4C). Univariate survival analysis of 51 patients, for whom follow-up information was available, revealed that MAGE-C1/CT7 and MAGE-C2/CT10 expression did not correlate with overall survival.

**Table 4:** Correlation between MAGE-C1/CT7, MAGE-C2/CT10 and Ki-67 expression in primary melanoma and risk of lymph node metastases.

A		
Variable	p value univariate	p value multivariate
**CT7 Positive**	0.001	0.005
**Ki-67 LI high**	0.03	0.29

B		
Variable	p value univariate	p value multivariate
**CT10 Positive**	<0.001	0.01
**Ki-67 LI high**	0.03	0.3

C		
Variable	p value univariate	p value multivariate
**CT7 Positive**	0.001	0.03
**CT10 Positive**	<0.001	0.01

The expression of MAGE-C1/CT7, MAGE-C2/CT10 and Ki-67 in primary melanoma was determined retrospectively and associated with the presence of SLN metastases. This analysis was performed either separately (univariate analysis) or together (multivariate analysis). The correlation was considered as significant by a p-value ≤0.05. The proportion of SLN metastases is significantly higher in patients with primary melanomas positive for MAGE-C1/CT7 and Ki-67 than in negative ones (univariate analysis). In a multivariate analysis, MAGE-C1/CT7 positivity is an independent predictor of metastasis when adjusted for Ki-67, whereas Ki-67 is not an independent predictor (p = 0.005 and p = 0.29 resp.) (A). The proportion of SLN metastases is significantly higher in patients with primary melanomas positive for MAGE-C2/CT10 (p<0.001); in a multivariate analysis MAGE-C2/CT10 positivity is an independent predictor of metastasis when adjusted for Ki-67, whereas Ki-67 is not an independent predictor (p = 0.01 and p = 0.3 resp.) (B). In a multivariate analysis expression of MAGE-C1/CT7 and MAGE-C2/CT10 remain independent predictors of SLN metastases also if adjusted for each other (p = 0.03 and p = 0.01 resp.) (C).

## Discussion

In this study we demonstrate the expression of MAGE-C1/CT7 and MAGE-C2/CT10 protein in primary melanoma and an increased expression in melanoma metastases. Furthermore, the expression of MAGE-C1/CT7 and/or MAGE-C2/CT10 in primary melanoma indicates a high risk of lymph node metastasis, which is of great clinical relevance as it identifies patients that require a strict follow-up.

MAGE-C1/CT7 and MAGE-C2/CT10 were co-expressed in more than 40% of melanoma lesions that were expressing either one of these two antigens. This observation is in accordance to other studies showing frequent co-expression of these and other CT antigens, justifying polyvalent CT-vaccine strategies in several tumor types [29]. MAGE-C1/CT7 was found in the nucleus as well as in the cytoplasm, while MAGE-C2/CT10 was present exclusively in the nucleus. This differential expression pattern is remarkable as these two antigens share a highly similar sequence of nearly 80% at the C-terminal region which contains the MAGE (melanoma antigen encoding gene) homologous sequence. MAGE-C1/CT7 has a unique feature in a form of repetitive sequence in the N-terminal region with an almost invariable core of 10 almost-exact repeats of 35 amino acid residues. Proteins with similar structure were shown to be involved in many physiological processes, such as cell cycle control, transcriptional regulation, cell signaling and apoptosis [30]. We recently investigated expression of MAGE-C1/CT7 in myeloma plasma cells from bone-marrow lesions of multiple myeloma patients and described that the sub-cellular localization of MAGE-C1/CT7 (nucleus or cytoplasm) has prognostic value such that combined nuclear and cytoplasmic expression correlates with reduced survival [27]. We could not confirm this correlation in melanoma due to the low number of lesions with an exclusive MAGE-C1/CT7 nuclear expression. Furthermore, a possible role of MAGE-C1/CT7 in promoting survival of myeloma cells through reduced apoptosis has been recently described [31]. Along the same line, we observed that MAGE-C1/CT7 and MAGE-C2/CT10 are more frequently expressed in melanoma metastases than in primary melanoma (20% versus 40%). This result is compatible with previous studies and supports a possible role for CT antigens in tumor progression [27], [32], [33], [34], [35] although these observations are difficult to confirm since the exact function of these antigens remains largely unknown. Further investigation of MAGE antigen function in these lesions might indeed provide fruitful insights in melanoma tumour biology.

We demonstrate that MAGE-C1/CT7 and MAGE-C2/CT10 expression in primary melanoma is a faithful predictor of lymph node metastasis in a patient cohort included in a recent study performed on tissue coming from sentinel lymph node biopsies from melanoma patients [24]. The SLN procedure is a technique in which, after tumor excision, the sentinel lymph node is removed and examined to determine whether malignant cells are present. This procedure has been successfully validated for melanoma as a mean of metastasis detection in patients with intermediate-thickness lesions [36]. Present state of the art of melanoma diagnosis includes Ki-67 staining as a prognostic marker [37], [38], [39], [40]. However, prediction of lymph node (LN) metastasis based on MAGE-C1/CT7 and MAGE-C2/CT10 expression in primary melanoma is independent of Ki-67 expression. Moreover, Ki-67 is not an independent predictive factor for metastatic spread if adjusted for MAGE-C1/CT7 or MAGE-C2/CT10, suggesting that these two CT antigens are superior markers to indicate lymph node metastasis. Our findings may have an important clinical impact because predictive markers of LN involvement are required for a better preoperative planning of the SLN procedure.

Expression of MAGE-C1/CT7 and MAGE-C2/CT10 in melanoma tissues was not correlated with overall patient survival which seems to be in contrast with the here reported results indicating a potential role in the tumorigenic process. This discrepancy might be due to the spontaneous immune response targeting these antigens which can block or down-regulate the tumor growth, as was previously shown for melanoma and several other cancers [41], [42]. Immunogenicity of MAGE-C2/CT10 has been previously demonstrated in melanoma and leukemia patients consisting of both cellular and humoral immune responses [18], [19], [20], [43], [44]. We recently showed spontaneous MAGE-C1/CT7-specific CD4^+^ T cells in the blood of patients with melanoma [16] and MAGE-C1/CT7-specific humoral response in multiple myeloma patients [23]. This immunogenicity taken together with frequent expression showed above strongly suggests that MAGE-C1/CT7 and MAGE-C2/CT10 are potential candidates for cancer immunotherapy in a large group of melanoma patients.

In conclusion, we found expression of MAGE-C2/CT10 and MAGE-C1/CT7 in a large proportion of melanoma patients, who could thus benefit from a specific anti-tumor immune response following vaccination with these antigens. Furthermore, our results identify the expression of MAGE-C1/CT7 and MAGE-C2/CT10 in the primary melanoma lesion as strong prediction markers for SLN metastases in melanoma patients; we therefore propose inclusion of these markers, together with Ki-67, to improve melanoma work-up.

## Supporting Information

Figure S1 **Antibody specificity by Western Blot.** Recombinant proteins (a) recombinant MAGE-C2/CT10 and (b) recombinant MAGE-C1/CT7) were detected by the corresponding monoclonal antibodies: anti-MAGE-C2/CT10 (A) and MAGE-C1/CT7-specific antibody (B). Degradation products can be seen in both cases.
(TIF)
