# Supplementary figures and images for: Expression of MAGE-C1/CT7 and MAGE-C2/CT10 Predicts Lymph Node Metastasis in Melanoma Patients
Source: PLoS One. 2011 Jun 27;6(6):e21418. doi: 10.1371/journal.pone.0021418 (PMC3124507; doi:10.1371/journal.pone.0021418)

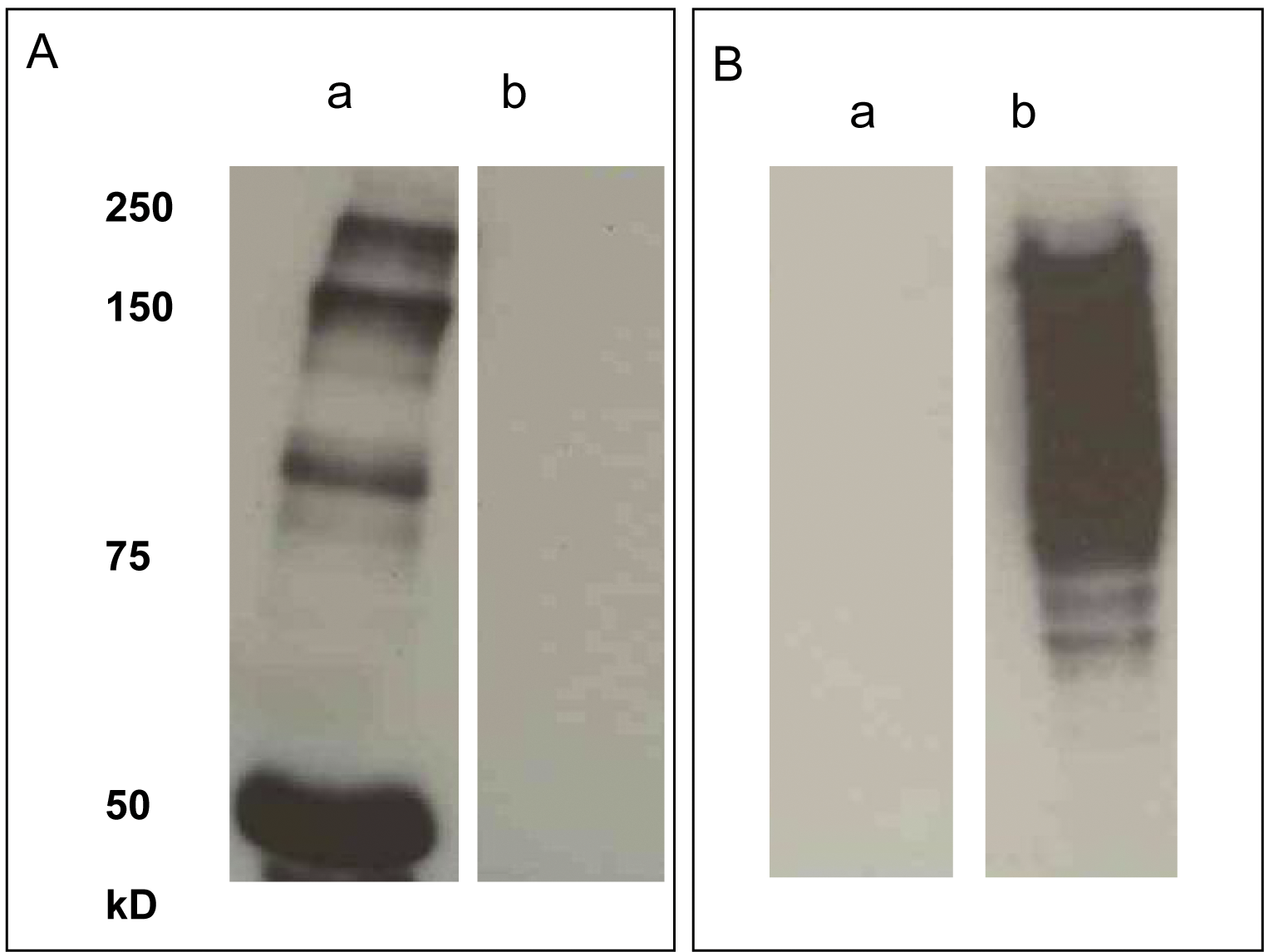

Supplement: Figure S1 — Antibody specificity by Western Blot. Recombinant proteins (a) recombinant MAGE-C2/CT10 and (b) recombinant MAGE-C1/CT7) were detected by the corresponding monoclonal antibodies: anti-MAGE-C2/CT10 (A) and MAGE-C1/CT7-specific antibody (B). Degradation products can be seen in both cases. (TIF) [file pone.0021418.s001.tif]
